# Supplementary material for: Replication fork binding triggers structural changes in the PriA helicase that govern DNA replication restart in E. coli
Source: Nat Commun. 2023 May 11;14:2725. doi: 10.1038/s41467-023-38144-x (PMC10175261; doi:10.1038/s41467-023-38144-x)
Supplement: Supplementary file 1 — Supplementary Information [file 41467_2023_38144_MOESM1_ESM.pdf]

**Replication fork binding triggers structural changes in the PriA helicase that govern DNA replication restart in *E. coli***

Alexander T. Duckworth[1], Peter L. Ducos[2,3,5], Sarah D. McMillan[1,5], Kenneth A. Satyshur[1], Katelien H. Blumenthal[1], Haley R. Deorio[1], Joseph A. Larson[1], Steven J. Sandler[4,*], Timothy Grant[2,3,*], and James L. Keck[1,*]

[1]Department of Biomolecular Chemistry, University of Wisconsin-Madison, Madison, Wisconsin, 53706, USA
[2]Department of Biochemistry, University of Wisconsin-Madison, Madison, Wisconsin, 53706, USA
[3]John and Jeanne Rowe Center for Research in Virology, Morgridge Institute for Research, Madison, Wisconsin, 53715, USA
[4]Department of Microbiology, University of Massachusetts at Amherst, Amherst, Massachusetts, 01003, USA
[5]These authors contributed equally to this work
*Correspondence: sandler@microbio.umass.edu (SJS), tim.grant@wisc.edu (TG), jlkeck@wisc.edu (JLK)

**Supplementary Table 1. Oligonucleotides used to construct synthetic replication forks**

| Name | Sequence (5′-3′) | Substrate name | Source |
|---|---|---|---|
| 1b-98 | GCA AGC CTT CTA CAG GTC GAC CGT CCA TGG CGA CTC GAG ACC GCA ATA CGG ATA AGG GCT GAG CAC GCC GAC GAA CAT TCA CCA CGC CAG ACC ACG TA | Forks 1 and 2 - Leading parental strand | 1 (Main text reference 62) |
| 3L-98 | GAC TAT CTA CGT CCG AGG CTC GCG CCG CAG ACT CAT TTA GCC CTT ATC CGT ATT GCG GTC TCG AGT CGC CAT GGA CGG TCG ACC TGT AGA AGG CTT GC | Forks 1 and 2 - Lagging parental strand | 1 |
| 11b-38 | TAC GTG GTC TGG CGT GGT GAA TGT TCG TCG GCG TGC TC | Forks 1 and 2 - Leading nascent strand | 1 |
| b-33 | AGT CTG CGG CGC GAG CCT CGG ACG TAG ATA GTC | Fork 2 - Lagging nascent strand | 1 |
| oTW140 | GCA AGC CTT CTA CAG GTC GAC CGT CCA TGG CGA CTC GAG ACC GCA ATA CGG ATA AGG GCT GAG CAC GCC GAC GAA CAT TCA CCA CGC CAG ACC ACG TAG TGC TAT AAG CTT CGC TGA CGT | Forks 3 and 4 - Extended 1b-98 | 2 (Main text reference 40) |
| oTW141 | AGC GTA ACG GAT CGA CCG CTT TGA CTA TCT ACG TCC GAG GCT CGC GCC GCA GAC TCA TTT AGC CCT TAT CCG TAT TGC GGT CTC GAG TCG CCA TGG ACG GTC GAC CTG TAG AAG GCT TGC | Forks 3 and 4 - Extended 3L-98 | 2 |
| oTW168 | ACG TCA GCG AAG CTT ATA GCA CTA CGT GGT CTG GCG TGG TGA ATG TTC GTC GGC GTG CTC | Forks 3 and 4 - Extended 11b-38 | 2 |
| oTW143 | AGT CTG CGG CGC GAG CCT CGG ACG TAG ATA GTC AAA GCG GTC GAT CCG TTA CGC T | Fork 4 – Extended b-33 | 2 |
| oTW144 | GCA AGC CTT CTA CAG GTC G | Primer for oTW141 for primer extension assay | 2 |
| oAD027 | CGA GAC CGC AAT ACG GAT AAG GGC TGA GCA CGC CGA CGA A | Fork 5 - Shortened 1b-98 for EM substrate | This work |
| oAD028 | GCC GCA GAC TCA TTT AGC CCT TAT CCG TAT TGC GGT CTC G | Fork 5 - Shortened 3L-98 for EM substrate | This work |
| oAD029 | TTC GTC GGC GTG CTC | Fork 5 - Shortened 11b-38 for EM substrate | This work |

## Supplementary Table 2. Cryo-EM data collection and model statistics

| **Data collection and processing** | **PriA/B-DNA (EMD-28959, PDB-8FAK)** |
|---|---|
| Microscope | Krios G3 |
| Voltage (kV) | 300 |
| Detector | K3 (Counting) / Falcon3 (Integrating) |
| Magnification (nominal/calibrated) | 81,000 (K3) / 96,000 (Falcon 3) |
| Data acquisition software | SerialEM |
| Electron exposure ($e^-/Å^2$) | 100 (K3) / 60 (Falcon 3) |
| Exposure rate (e-/pixel per s) | 6.83 (K3) |
| Number of frames per micrograph | 102 (K3) / 60 (Falcon 3) |
| Pixel size (Å) | 1.079 (K3) / 0.8333 (Falcon 3) |
| Defocus range (µm) | -0.5 to -2.5 |
| Micrographs collected (no.) | 1595 (K3) / 1137 (Falcon 3) |
| **Reconstruction** | |
| Image-processing package | cisTEM |
| Total extracted particles (no.) | 1,539,320 |
| Final particles (no.) | 186,058 |
| Symmetry imposed | C1 |
| PartFSC 0.143 global resolution (Å) | 3.2 |
| **Model composition** | |
| Protein Residues | 837 |
| Nucleotide Residues | 57 |
| Ligands | 2 |
| **Refinement** | |
| Refinement package | Phenix |
| CC (volume/mask) | 0.74/0.74 |
| R.m.s deviations | |
| Bond lengths (Å) | 0.002 |
| Bond angles (°) | 0.461 |
| **Validation** | |
| Map-to-model FSC 0.5 | 3.6 |
| Ramachandran plot | |
| Outliers | 0 |
| Allowed (%) | 2.3 |
| Favored (%) | 97.7 |
| MolProbity score | 1.53 |
| Poor rotamers (%) | 0 |
| Clashscore (all atoms) | 8.55 |
| C-beta deviations (%) | 0 |
| CaBLAM Outliers (%) | 2.69 |

**Supplementary Table 3. The effect of *priA*, *priB* and *priC* mutations on cell size**

| | SS6321 | SS13598 | SS9116 | SS13914 | SS13901 | SS13913 |
|---|---|---|---|---|---|---|
| ***priA*** | + | *tm* | + | + | *tm* | *tm* |
| ***priB*** | + | + | Δ | + | Δ | + |
| ***priC*** | + | + | + | *null* | + | *null* |
| **Avg. Cell Area** | 715 | 760 | 774 | 797 | 793 | 1297 |
| | Frequencies of cells within a particular size range | | | | | |
| **0 ≤ 600** | 35.6 | 24.5 | 25.8 | 19.0 | 18.6 | 12.3 |
| **601 ≤ 700** | 21.8 | 20.2 | 20.7 | 20.9 | 19.8 | 12.0 |
| **701 ≤ 800** | 14.1 | 19.3 | 17.7 | 18.5 | 20.0 | 10.5 |
| **801 ≤ 900** | 10.3 | 14.4 | 11.3 | 12.9 | 15.8 | 10.2 |
| **901 ≤ 1000** | 7.8 | 8.6 | 9.5 | 9.8 | 10.5 | 9.9 |
| **1001 ≤ 1100** | 5.7 | 6.4 | 6.3 | 8.5 | 7.3 | 8.3 |
| **1101 ≤ 1200** | 2.7 | 3.4 | 3.8 | 5.2 | 3.9 | 7.9 |
| **1201 ≤ 1300** | 0.7 | 1.1 | 2.2 | 2.3 | 1.8 | 6.3 |
| **1301 ≤ 1400** | 0.4 | 0.4 | 0.6 | 1.5 | 0.9 | 1.9 |
| **1401 ≤ 1500** | 0.1 | 0.7 | 0.6 | 0.4 | 0.8 | 2.5 |
| **1501 ≤ 1600** | 0.2 | 0.3 | 0.3 | 0.6 | 0.2 | 2.5 |
| **1601 ≤ 4000** | 0.5 | 0.7 | 1.4 | 0.4 | 0.5 | 15.7 |

**Supplementary Table 4. The effect of *priA*, *priB* and *priC* mutations on SOS expression measured via relative fluorescence intensity**

| | SS6321 | SS13598 | SS9116 | SS13914 | SS13901 | SS13913 |
|---|---|---|---|---|---|---|
| ***priA*** | + | *tm* | + | + | *tm* | *tm* |
| ***priB*** | + | + | Δ | + | Δ | + |
| ***priC*** | + | + | + | *null* | + | *null* |
| **Avg. Rel. Intensity** | 1.23 | 1.00 | 1.09 | 1.16 | 1.01 | 1.97 |
| | Frequencies of cells within a particular intensity range | | | | | |
| **0 ≤ 1** | 39.2 | 67.1 | 55.4 | 47.5 | 59.1 | 21.4 |
| **1.1 ≤ 2** | 56.8 | 28.6 | 40.0 | 48.6 | 39.1 | 48.1 |
| **2.1 ≤ 3** | 2.5 | 2.3 | 3.3 | 1.8 | 1.3 | 15.4 |
| **3.1 ≤ 4** | 0.2 | 1.0 | 0.1 | 0.6 | 0.3 | 7.1 |
| **4.1 ≤ 5** | 0.5 | 0.3 | 0.3 | 0.6 | 0.0 | 2.8 |
| **5.1 ≤ 6** | 0.1 | 0.2 | 0.2 | 0.1 | 0.1 | 2.2 |
| **6.1 ≤ 7** | 0.0 | 0.1 | 0.2 | 0.1 | 0.1 | 0.9 |
| **7.1 ≤ 8** | 0.0 | 0.2 | 0.1 | 0.2 | 0.0 | 0.7 |
| **8.1 ≤ 9** | 0.1 | 0.0 | 0.1 | 0.2 | 0.0 | 0.4 |
| **9.1 ≤ 10** | 0.1 | 0.1 | 0.1 | 0.1 | 0.0 | 0.3 |
| **10.1 ≤ 11** | 0.0 | 0.1 | 0.0 | 0.0 | 0.1 | 0.3 |
| **11.1 ≤ 30** | 0.3 | 0.1 | 0.1 | 0.3 | 0.0 | 0.3 |

## Supplementary Table 5. Strain list

| Strain | *priA* | other relevant genotype | Source or derivation |
|---|---|---|---|
| JC19281 | | *zba-3055::Tn10 priC303::kan* | 3 (Main text reference 52) |
| SS6321[a] | + | *hupA::mcherry del(attB)::sulAp-gfp* | 4 |
| SS6796 | | *zjf-599::Tn10 del(priB)302* | 4 |
| SS9116 | | *del(priB)302 hupA::mcherry del(attB)::sulAp-gfp* | 4 |
| SS9276 | *del(priA)317::kan* | *dnaC809,820* | 5 (Main text reference 47) |
| SS13594 | *355::cat* | | This study |
| SS13598 | *355::cat* | *hupA::mcherry del(attB)::sulAp-gfp* | SS13594 → SS6321[b] |
| SS13901 | *355::cat* | *zjf-599::Tn10 del(priB)302 hupA::mcherry del(attB)::sulAp-gfp* | SS6796 → SS13598[c] |
| SS13913 | *355::cat* | *zba-3055::Tn10 priC303::kan hupA::mcherry del(attB)::sulAp-gfp* | JC19281 → SS13598[d] |
| SS13914 | | *zba-3055::Tn10 priC303::kan hupA::mcherry del(attB)::sulAp-gfp* | JC19281 → SS6321[d] |

[a] SS6321 partial genotype is: *del(lacMS286) attphi80::phi80::del(lacBK1) argE3 his-4 thi-1 rpsL31 xyl-5 mtl-1 tsx sulB103*. It is a derivative of SK362[6].
[b] Select chloramphenicol resistance screen for *priA355* with PCR and restricting with *Bst* XI.
[c] Select tetracycline resistance on minimal media and screen *del(priB)302* by PCR.
[d] Select tetracycline resistance on rich media and screen for kanamycin resistance.

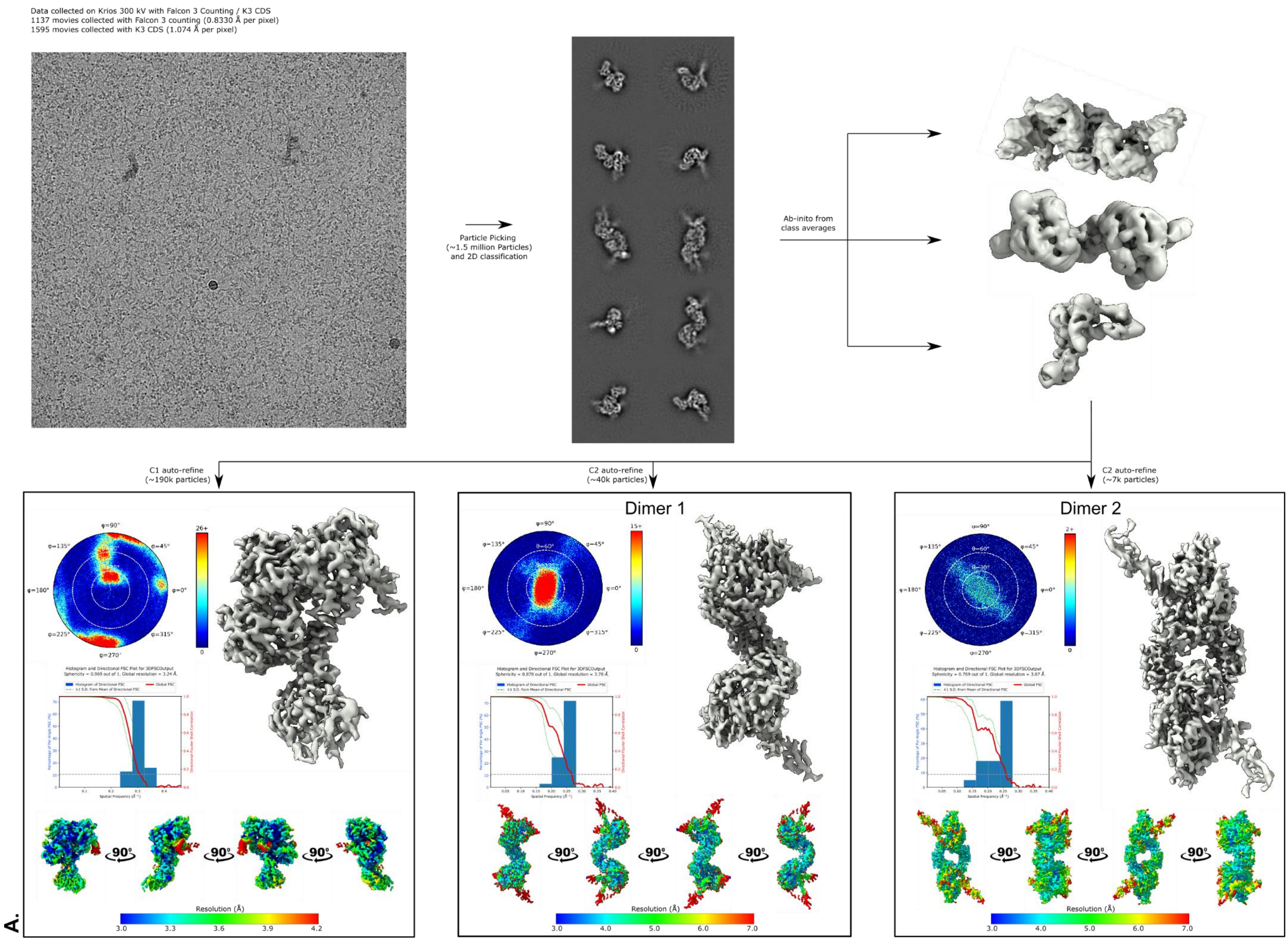
Data collected on Krios 300 kV with Falcon 3 Counting / K3 CDS
1137 movies collected with Falcon 3 counting (0.8330 Å per pixel)
1595 movies collected with K3 CDS (1.074 Å per pixel)
Particle Picking
(~1.5 million Particles)
and 2D classification
Ab-inito from
class averages
C1 auto-refine
(~190k particles)
C2 auto-refine
(~40k particles)
C2 auto-refine
(~7k particles)
Dimer 1
Dimer 2
Resolution (Å)
90°
A.

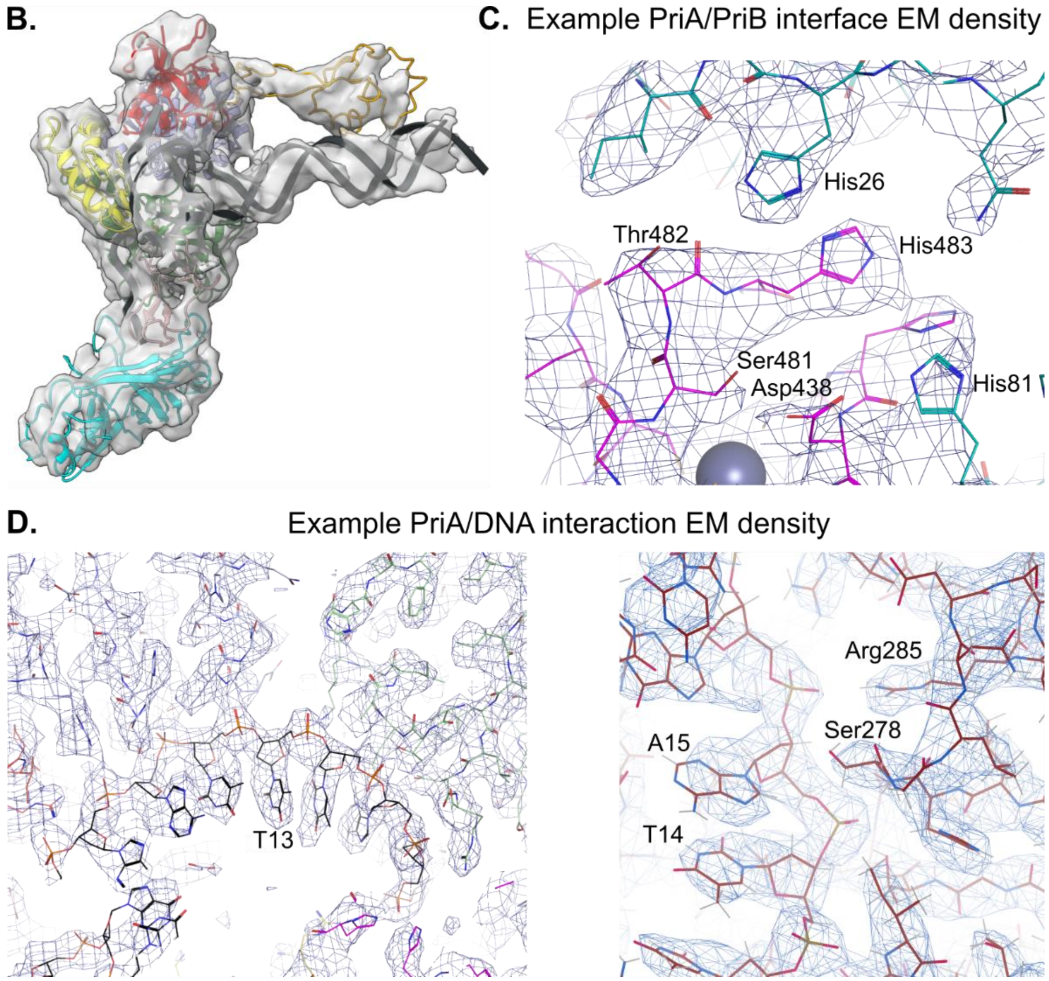


**Supplementary Figure 1. Workflow for cryo-EM structure determination and resulting characteristic density maps. (A)** Graphical description of cryo-EM processing methods used in this study. The workflow demonstrates a typical image and representative good class averages. Each of the final 3 reconstructions is shown alongside the angular plot demonstrating the distribution of particle views and the 3D Fourier shell correlation[7]. Also shown is the local resolution volume in a number of different orientations. **(B)** Result of focused classification with focus on the extended parental DNA strand and PriA-WH. Views of the molecular model with associated cryo-EM density map. **(C)** Model of the PriA/PriB interacting region overlayed with EM density map (colored as in Figure 1). **(D)** Two views of the PriA/DNA interacting region overlayed with EM density map.

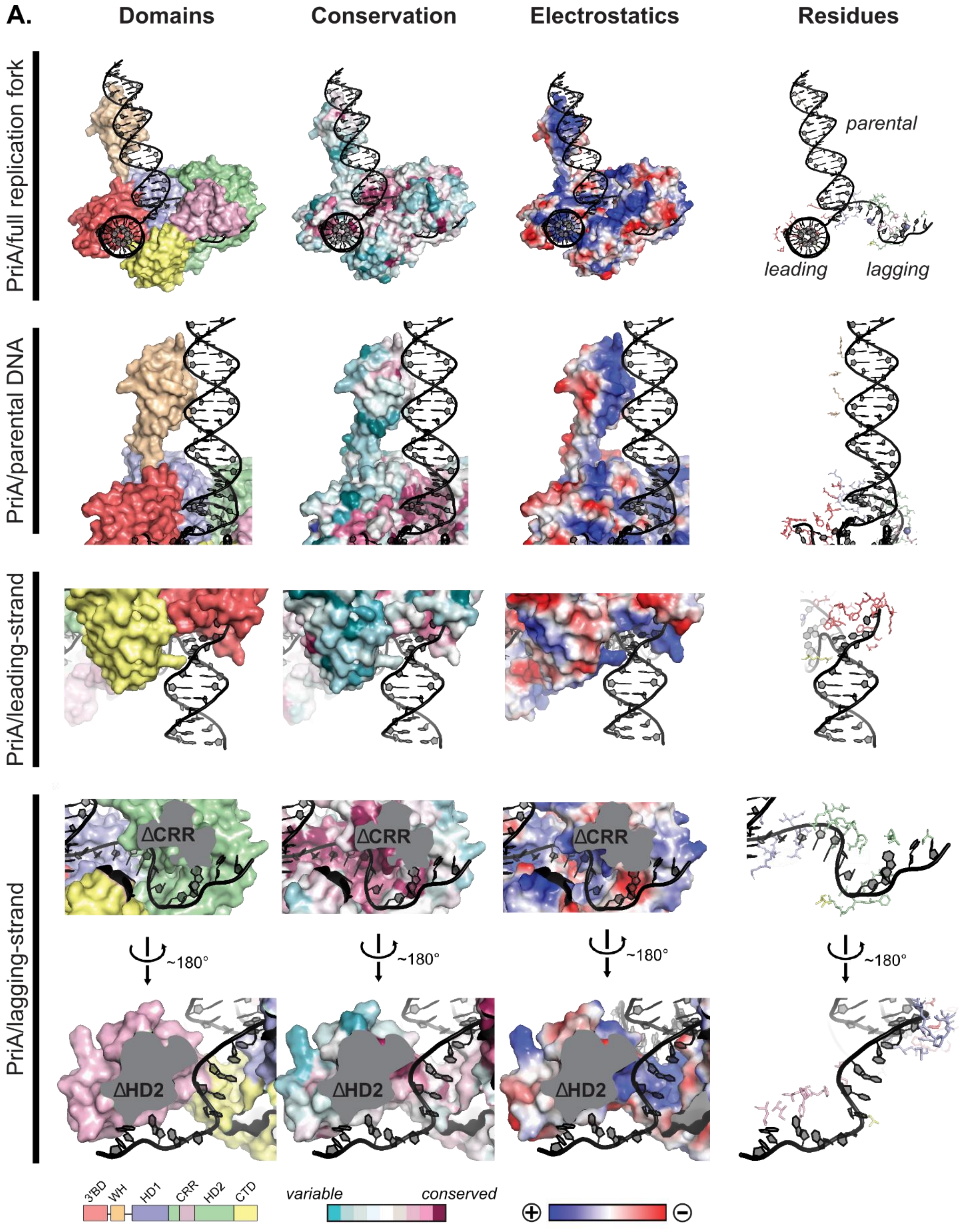

A.
Domains
Conservation
Electrostatics
Residues
PriA/full replication fork
PriA/parental DNA
PriA/leading-strand
PriA/lagging-strand
parental
leading
lagging
ΔCRR
~180°
ΔHD2
3'BD
WH
HD1
CRR
HD2
CTD
variable
conserved

B.

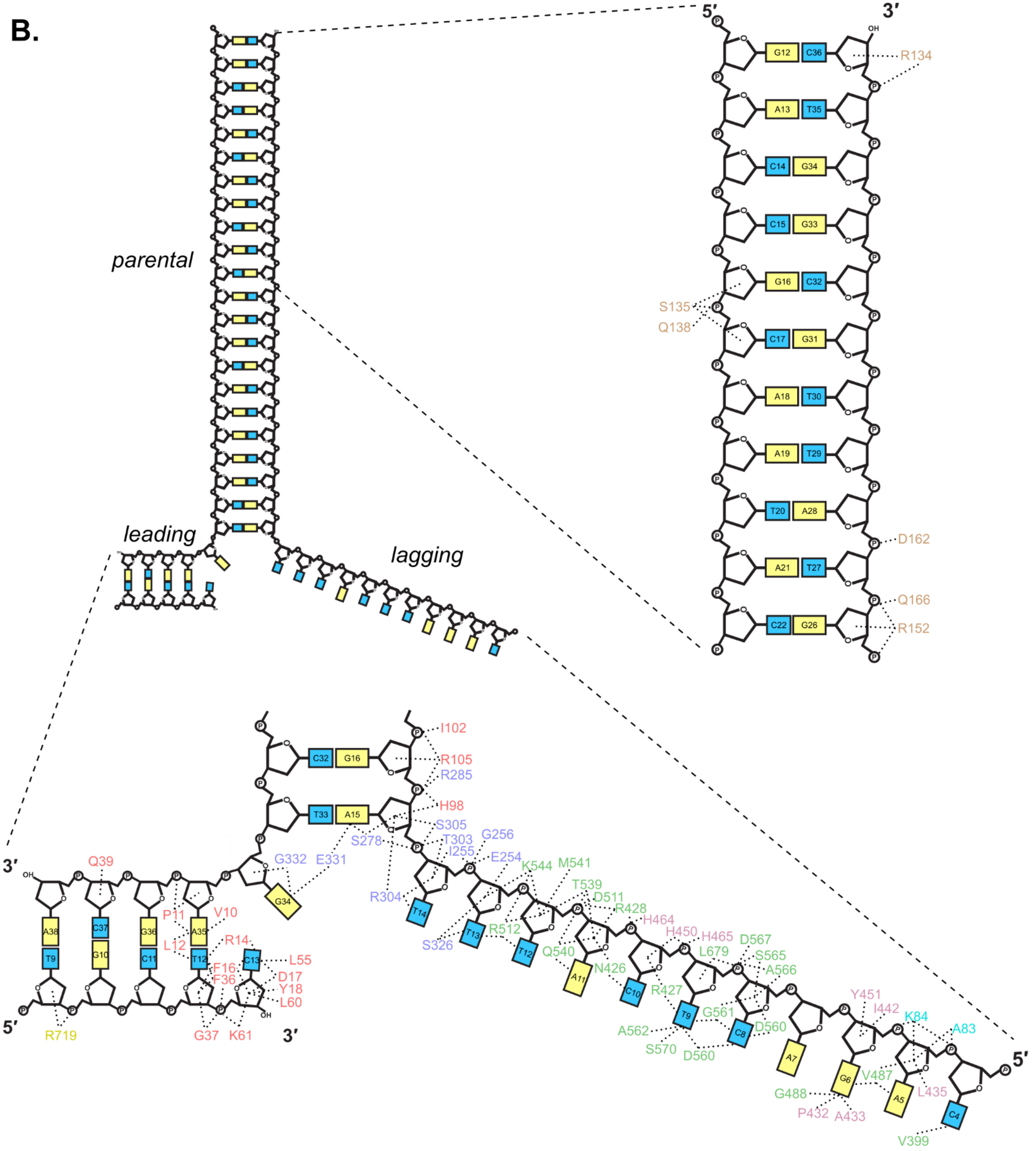

parental
leading
lagging
5′
3′
OH
G12
C36
R134
A13
T35
C14
G34
C15
G33
G16
C32
S135
Q138
C17
G31
A18
T30
A19
T29
T20
A28
D162
A21
T27
Q166
C22
G26
R152
I102
C32
G16
R105
R285
H98
T33
A15
S305
S278
T303
G256
I255
E254
G332
E331
K544
M541
T539
D511
R428
H464
H450
H465
D567
S565
A566
L679
R304
T14
S326
T13
R512
T12
Q540
A11
N426
C10
R427
T9
G561
C8
D560
A562
S570
D560
A7
Y451
I442
K84
A83
G6
V487
A5
L435
G488
P432
A433
C4
V399
Q39
A38
T9
C37
G10
G36
C11
A35
T12
C13
P11
V10
L12
R14
F16
F36
L55
D17
Y18
L60
G34
R719
G37
K61

**Supplementary Figure 2. PriA/replication fork DNA contacts within the PriA/PriB/DNA structure. (A)** PriA/replication fork contacts, with PriB removed to better visualize PriA-specific interactions. PriA is colored to reflect the domain structure, evolutionary conservation, or electrostatic potential, respectively, in the first three columns. The fourth column shows specific residues that were identified as contacting DNA using the DNAproDB server[8] (Main text reference 39). Rows (top to bottom) show the PriA contact with the full DNA replication fork, parental DNA, leading-strand DNA, and lagging-strand DNA, respectively. Note that two views (rotated ~180° along the y-axis) are provided for the lagging-strand with grey surfaces showing where the CRR or HD2 have been removed from the model to allow visualization of ssDNA within the pore. **(B)**. Schematic of the replication fork with zoomed-in images showing PriA and PriB DNA-binding residues. Interactions with the nucleotide base, deoxyribose, or phosphate backbone are shown. Residue labels are colored by domain as shown in Figure 1B.

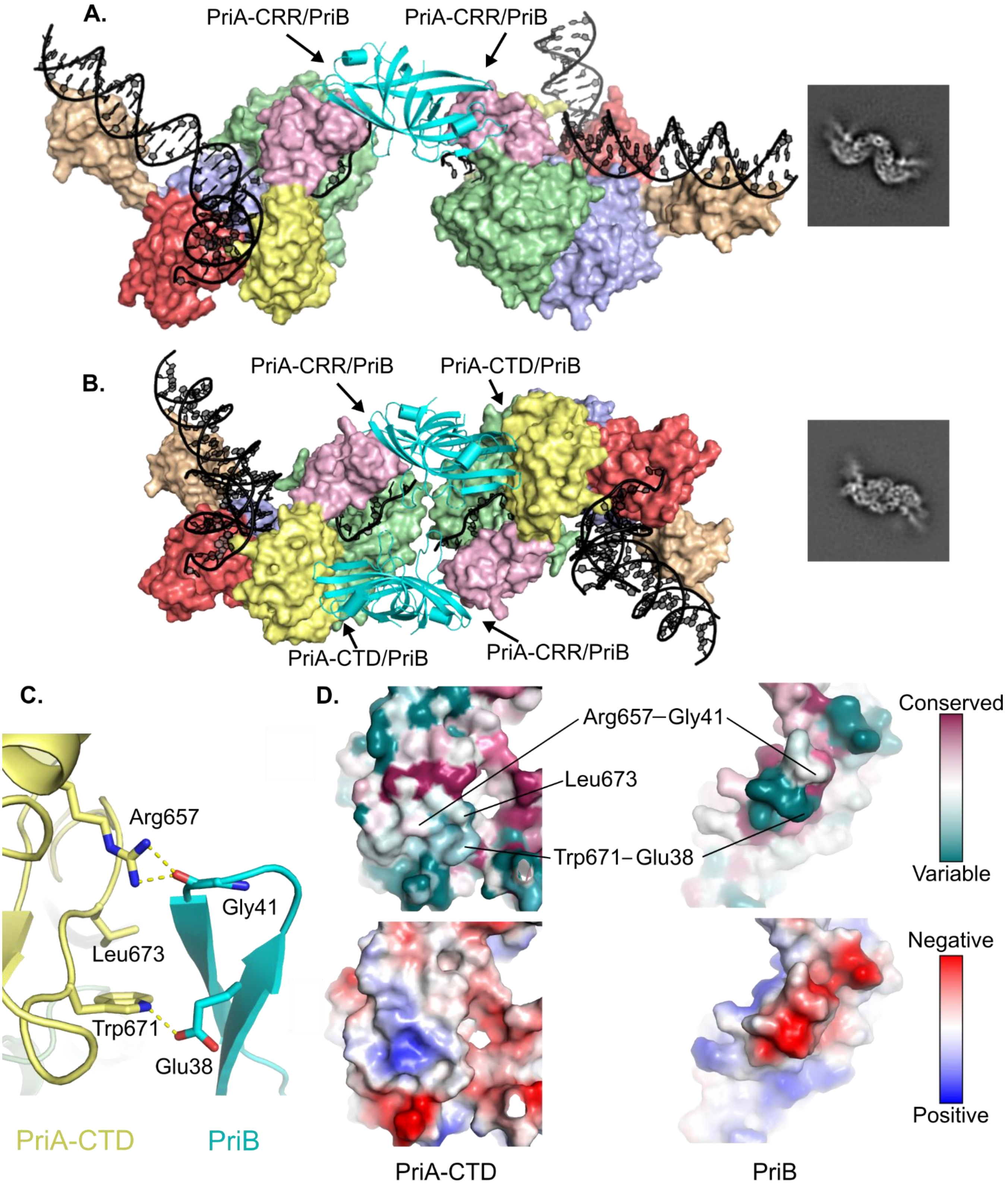

A.
PriA-CRR/PriB
PriA-CRR/PriB
B.
PriA-CRR/PriB
PriA-CTD/PriB
PriA-CTD/PriB
PriA-CRR/PriB
C.
Arg657
Gly41
Leu673
Trp671
Glu38
PriA-CTD
PriB
D.
Arg657–Gly41
Leu673
Trp671–Glu38
Conserved
Variable
Negative
Positive
PriA-CTD
PriB

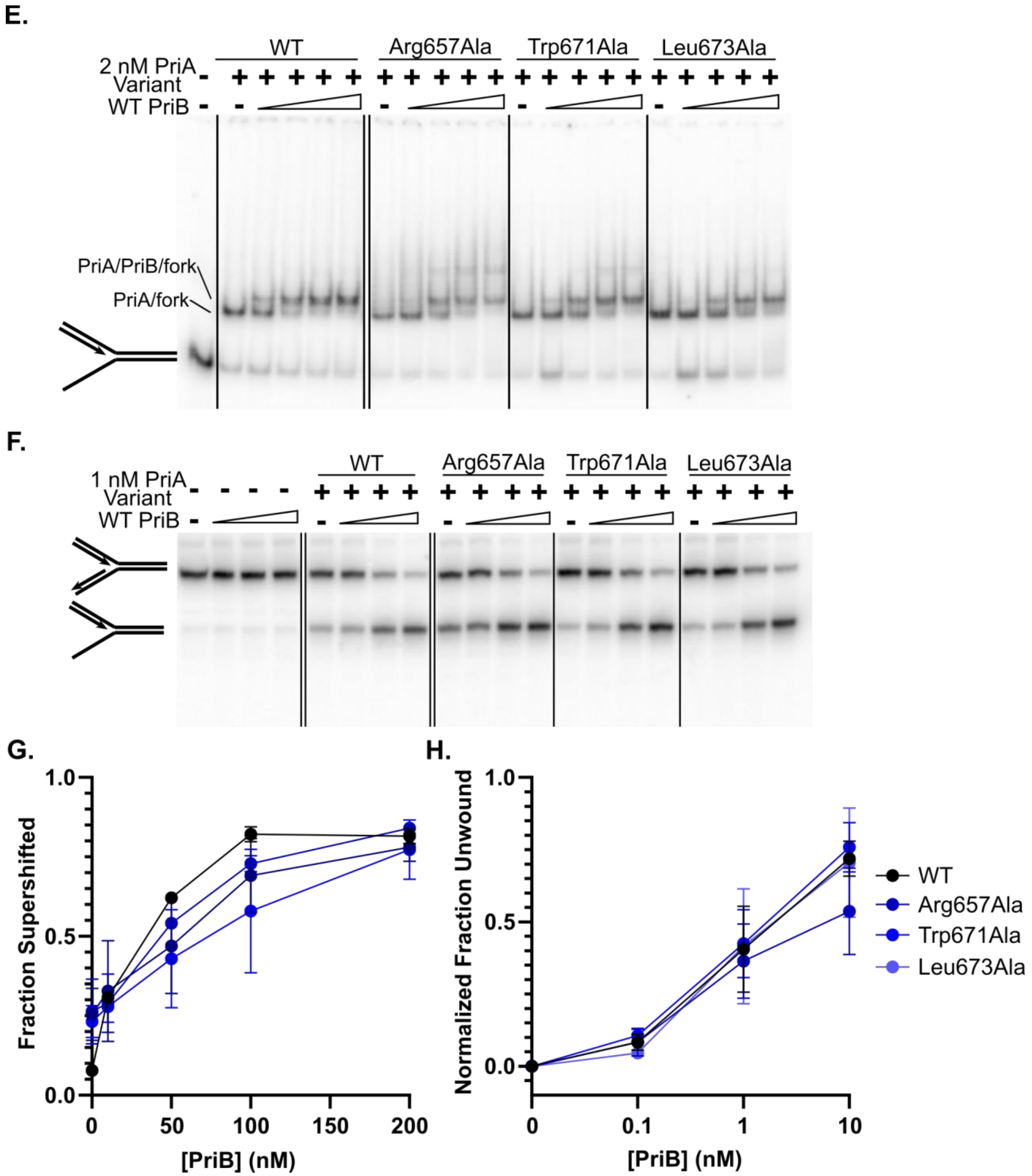
E.
WT
Arg657Ala
Trp671Ala
Leu673Ala
2 nM PriA Variant
WT PriB
PriA/PriB/fork
PriA/fork
F.
WT
Arg657Ala
Trp671Ala
Leu673Ala
1 nM PriA Variant
WT PriB
G.
Fraction Supershifted
[PriB] (nM)
H.
Normalized Fraction Unwound
[PriB] (nM)
WT
Arg657Ala
Trp671Ala
Leu673Ala

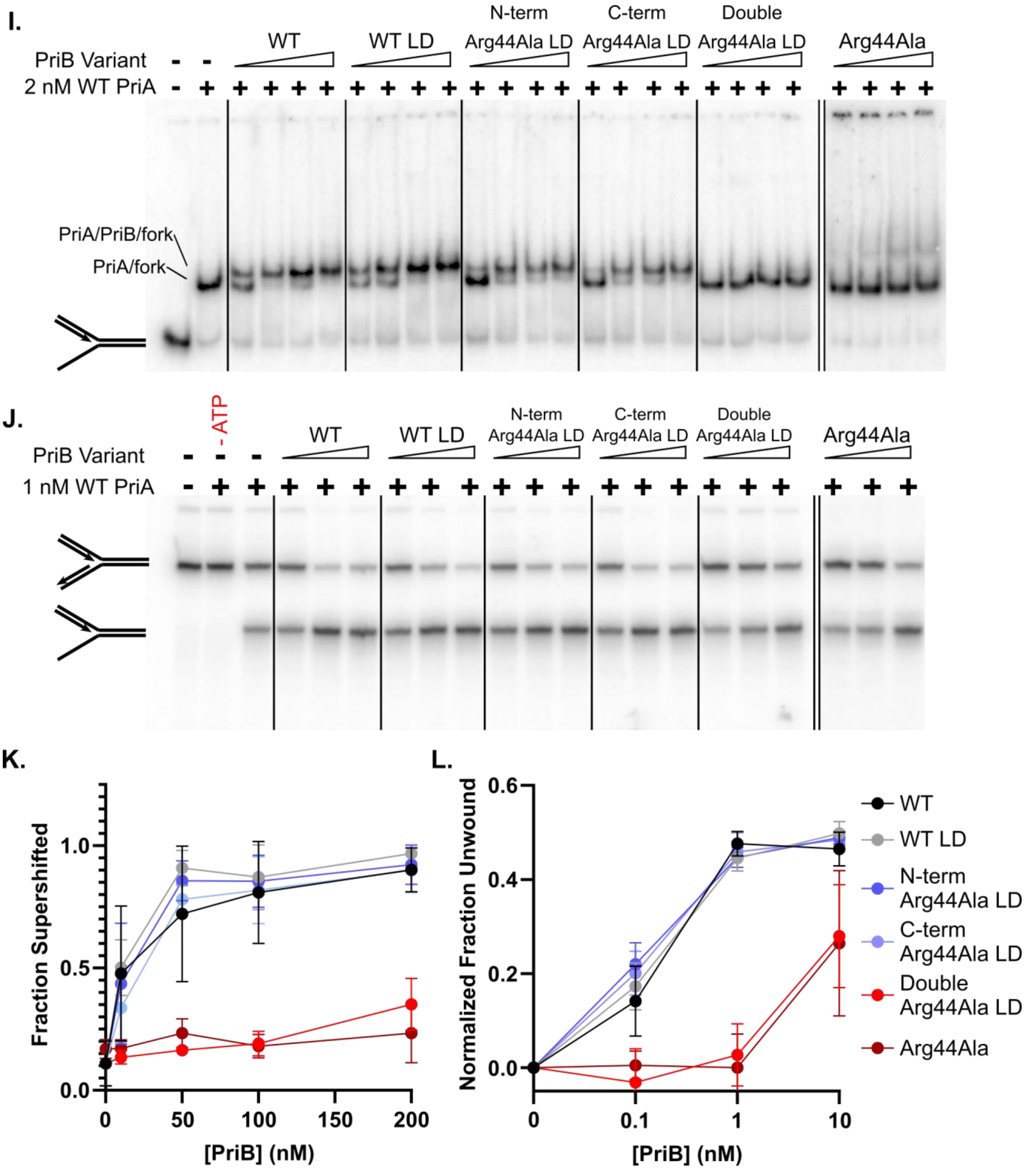

I.
PriB Variant
2 nM WT PriA
WT
WT LD
N-term Arg44Ala LD
C-term Arg44Ala LD
Double Arg44Ala LD
Arg44Ala
PriA/PriB/fork
PriA/fork
J.
- ATP
PriB Variant
1 nM WT PriA
WT
WT LD
N-term Arg44Ala LD
C-term Arg44Ala LD
Double Arg44Ala LD
Arg44Ala
K.
Fraction Supershifted
[PriB] (nM)
0.0
0.5
1.0
0
50
100
150
200
L.
Normalized Fraction Unwound
[PriB] (nM)
0.0
0.2
0.4
0.6
0
0.1
1
10
WT
WT LD
N-term Arg44Ala LD
C-term Arg44Ala LD
Double Arg44Ala LD
Arg44Ala

**Supplementary Figure 3. Alternative PriA/PriB/replication fork structures found using EM are not relevant *in vitro*. (A)** Dimer 1 structure where PriA/replication fork complexes are bound to both sides of a PriB dimer (cyan) via the PriA-CRR (magenta). A representative 2D class average is shown. **(B)** Dimer 2 structure showing PriA/replication fork complexes bound to both sides of two PriB dimers (cyan) via the PriA-CRR (magenta) and PriA-CTD (yellow). A representative 2D class average is shown. **(C)** Residues within the PriA-CTD/PriB interface that are within hydrogen bonding distance. **(D)** Surface representations of the PriA-CTD/PriB interface colored by conservation (top) or electrostatic surface potential (bottom). Interacting residues are highlighted. Conservation status was calculated using the ConSurf server[9] (Main text reference 73). As PriB is not well conserved across bacteria[10] (Main text reference 33), the calculation of PriA conservation was limited to 150 sequences from species containing a *priB* gene. **(E)** EMSAs of PriA-CTD variants binding to wild-type PriB. **(F)** PriB stimulation of PriA-CTD variant helicase activity. **(G)** Quantification of the ratio of PriB-supershifted (PriA/PriB/DNA complexes) to PriA-shifted (PriA/DNA) bands in (E). **(H)** Quantification of percentage unwound normalized to 0 nM PriB reactions in (F). **(I)** EMSAs of PriB LD variants binding to wild-type PriA. **(J)** PriB LD stimulation of PriA helicase activity. **(K)** Quantification of the ratio of PriB-supershifted (PriA/PriB/DNA complexes) to PriA-shifted (PriA/DNA) bands in (I). **(L)** Quantification of percentage unwound normalized to 0 nM PriB reactions in (J). Data points in all plots are the mean of three measurements +/- standard deviation. Uncropped images and quantification data for all replicates are provided in the Source Data file.

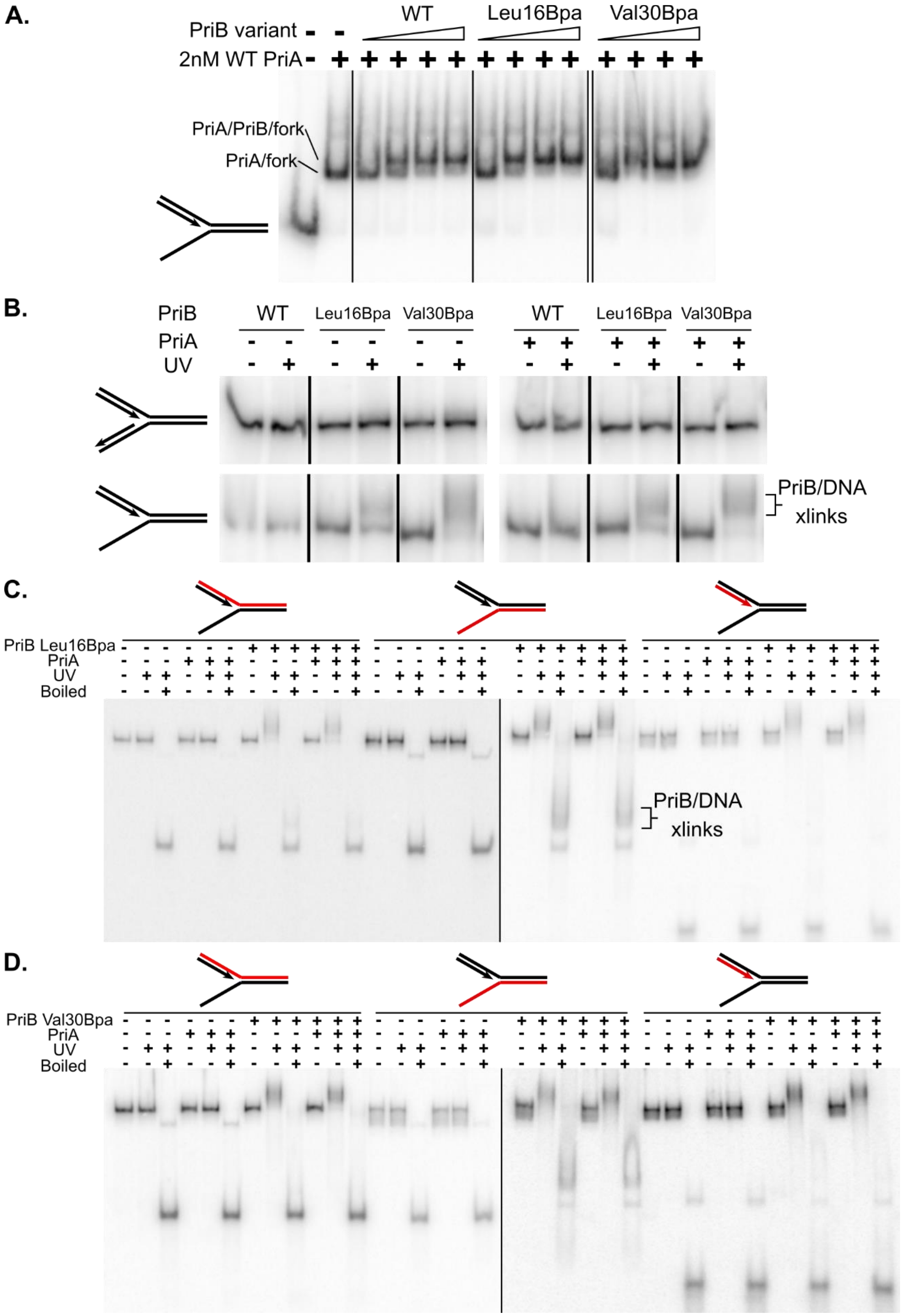

A.
PriB variant
WT
Leu16Bpa
Val30Bpa
2nM WT PriA
PriA/PriB/fork
PriA/fork
B.
PriB
PriA
UV
WT
Leu16Bpa
Val30Bpa
PriB/DNA xlinks
C.
PriB Leu16Bpa
PriA
UV
Boiled
PriB/DNA xlinks
D.
PriB Val30Bpa
PriA
UV
Boiled

**Supplementary Figure 4. PriB Bpa variants retain wild-type functionality and crosslink predominantly to ssDNA lagging strand in DNA replication forks. (A)** EMSAs assessing PriB Bpa variant binding to PriA/DNA complexes. **(B)** PriB Bpa variants crosslinked to Fork 4 (top gels) or Fork 3 (bottom gels) in the presence and absence of PriA. **(C and D)** PriB L16Bpa **(C)** or PriB V30Bpa **(D)** were crosslinked to versions of Fork 3 with each strand labeled. Boiling the reactions after crosslinking reveals strand specific crosslinks. Radiolabeled strand is indicated in red. Gels in panels A, C, and D are representative of three replicate experiments. Panel B was performed once. Uncropped images of all replicates are provided in the Source Data file.

**A.**

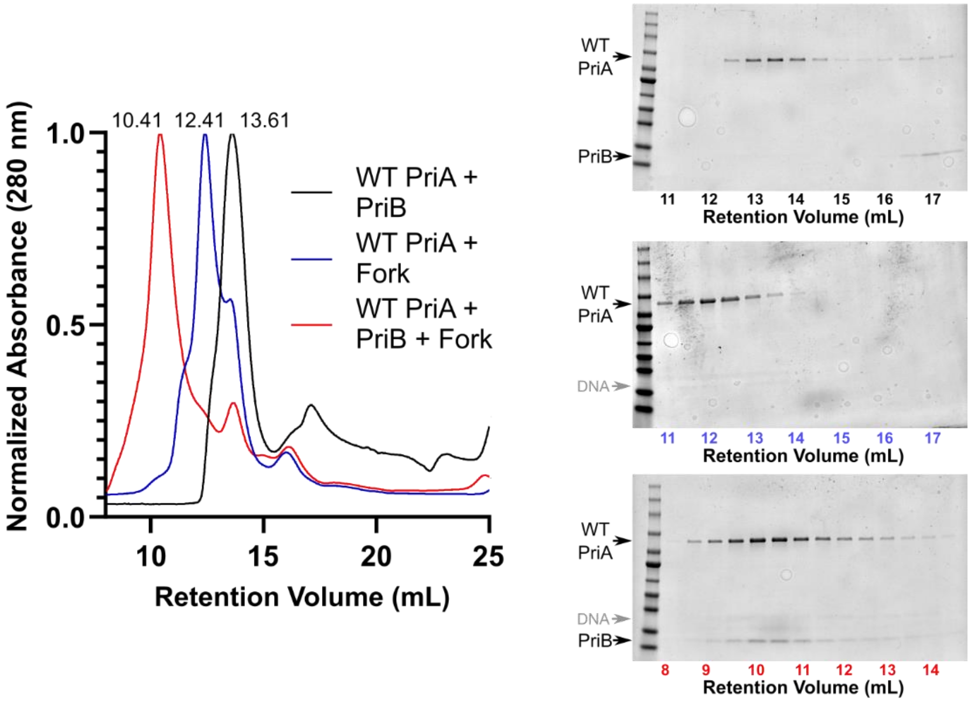
10.41
12.41
13.61
1.0
0.5
0.0
Normalized Absorbance (280 nm)
10
15
20
25
Retention Volume (mL)
WT PriA + PriB
WT PriA + Fork
WT PriA + PriB + Fork
WT PriA
PriB
11 12 13 14 15 16 17
Retention Volume (mL)
WT PriA
DNA
11 12 13 14 15 16 17
Retention Volume (mL)
WT PriA
DNA
PriB
8 9 10 11 12 13 14
Retention Volume (mL)

**B.**

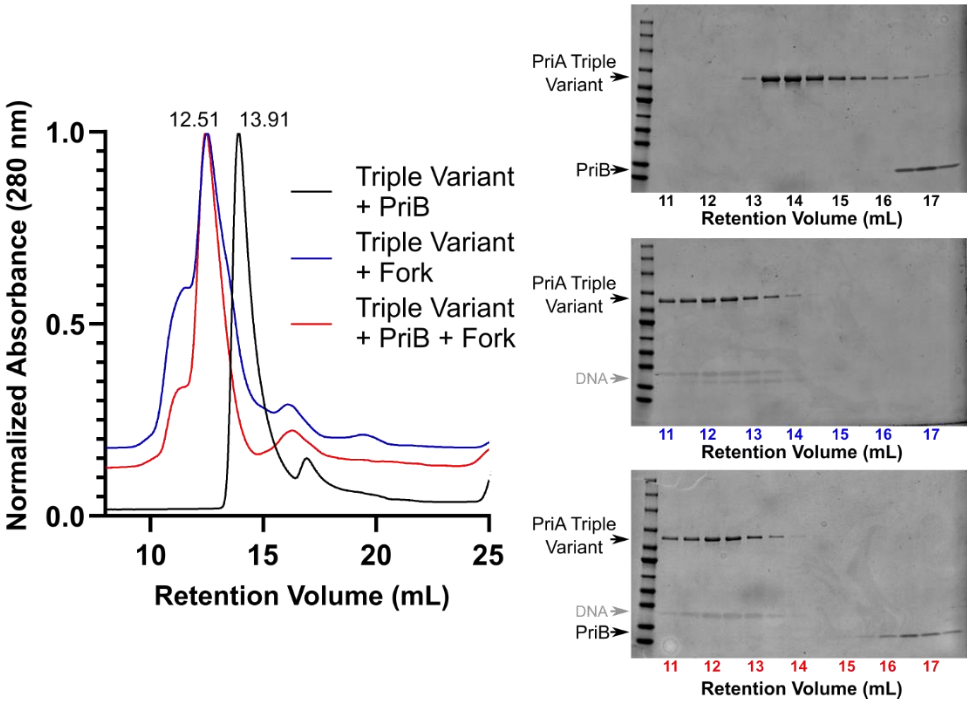
12.51
13.91
1.0
0.5
0.0
Normalized Absorbance (280 nm)
10
15
20
25
Retention Volume (mL)
Triple Variant + PriB
Triple Variant + Fork
Triple Variant + PriB + Fork
PriA Triple Variant
PriB
11 12 13 14 15 16 17
Retention Volume (mL)
PriA Triple Variant
DNA
11 12 13 14 15 16 17
Retention Volume (mL)
PriA Triple Variant
DNA
PriB
11 12 13 14 15 16 17
Retention Volume (mL)

**Supplementary Figure 5. PriB does not coelute with PriA-CRR triple variant. (A)** Size-exclusion chromatography profile of wild-type PriA combined with PriB (black), with Fork 5 (blue), or with both (red). SDS-PAGE gels of the resulting fractions are shown (upper gel – PriA and PriB, middle gel – PriA and Fork 5, lower gel – PriA, PriB, and Fork 5). Note: Bottom gel samples were taken from earlier retention volumes to accommodate the peak shift. **(B)** Size-exclusion chromatography profile of the PriA-CRR triple variant combined with PriB (black), Fork 5 (blue), or both (red). SDS-PAGE gels of the resulting fractions are shown (upper gel – PriA and PriB, middle gel – PriA and Fork 5, lower gel – PriA, PriB, and Fork 5). This experiment was done once. Uncropped gels are provided in the Source Data file. Molecular weights for the ladder are as follows from top to bottom (in kDa): 250, 180, 130, 95, 72, 55, 43, 34, 26, 17, 11.

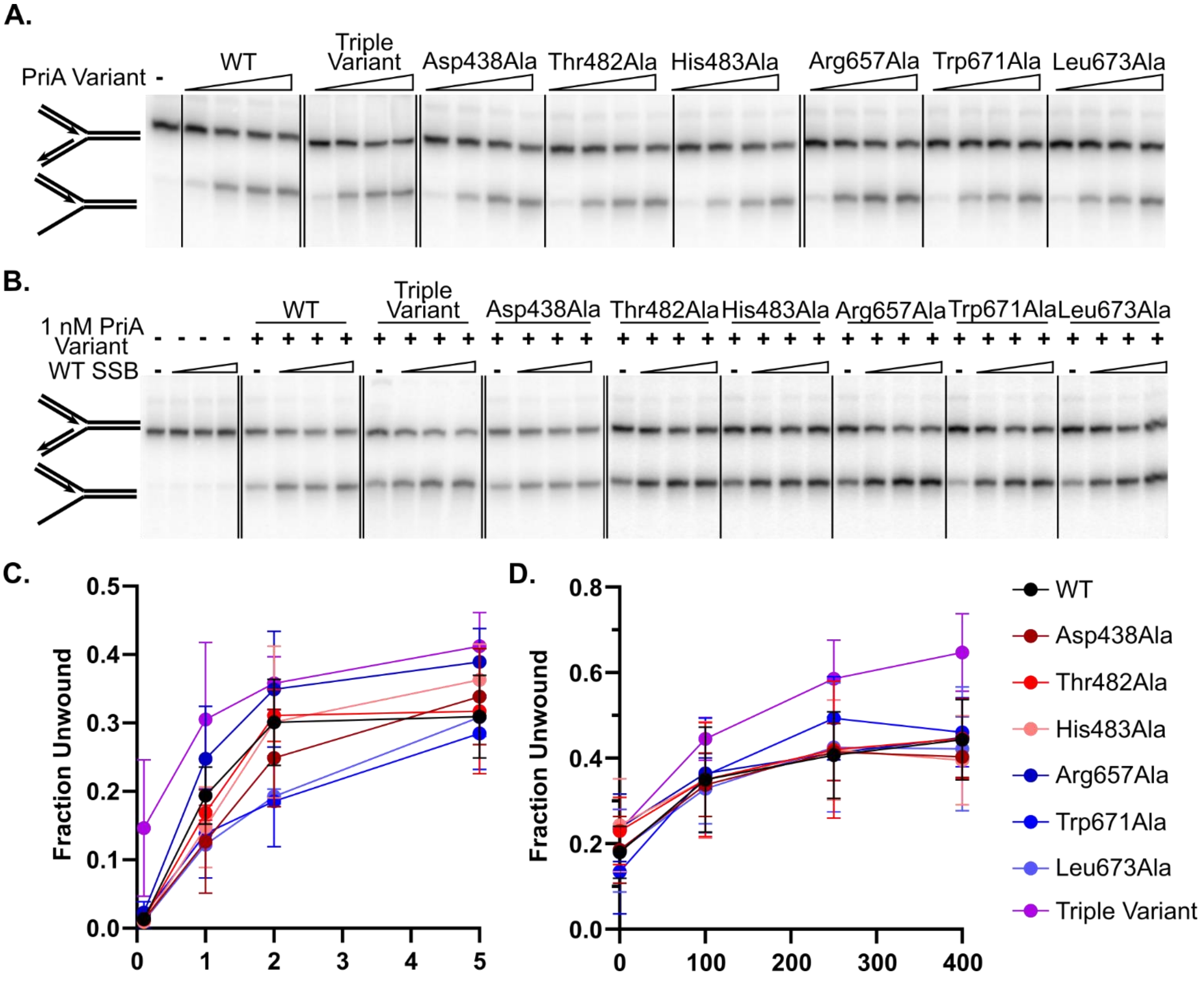


**Supplementary Figure 6. PriA-CRR and -CTD variants retain helicase activity and SSB-stimulation. (A)** Assessment of PriA variant helicase activity in the absence of stimulatory proteins (PriB or SSB). **(B)** SSB stimulation of PriA variant helicase activity assay. **(C and D)** Quantification of percentage unwound in **(A)** and **(B)**, respectively. Data points are the mean of three measurements +/- standard deviation. Uncropped images and quantification data for all replicates are provided in the Source Data file.

**A.**

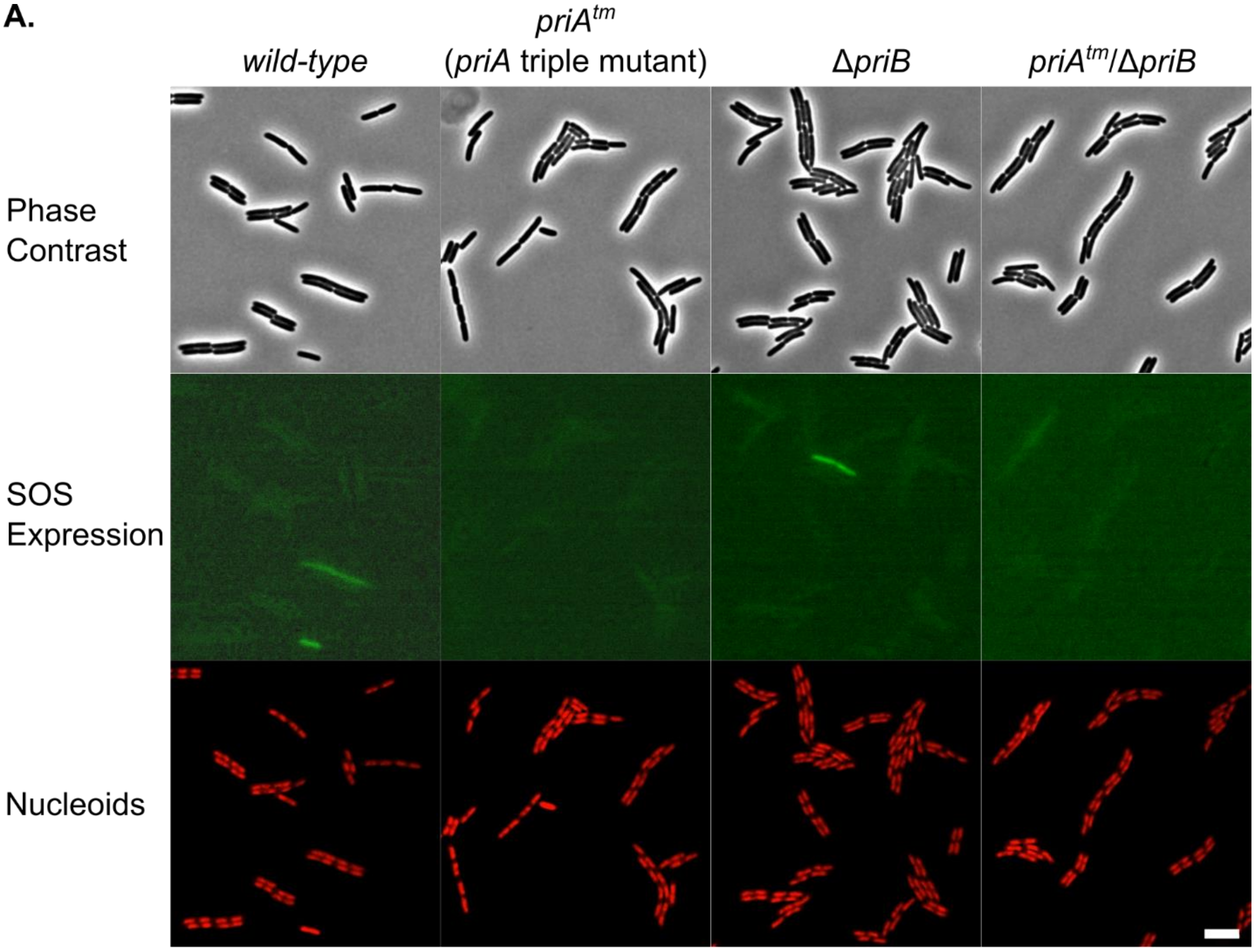


**B.**

| Strain | SS6321 | SS13598 | SS9116 | SS13901 |
|---|---|---|---|---|
| ***priA*** | + | *tm* | + | *tm* |
| ***priB*** | + | + | Δ | Δ |
| **Avg. Cell Area** | 715 | 760 | 774 | 793 |
| **Rel. SOS Intensity** | 1.23 | 1.00 | 1.09 | 1.01 |
| **Chromosome Partitioning** | + | + | + | + |

**Supplementary Figure 7. Pairing a Δ*priB* mutation with *priA*$^{tm}$ causes no cellular defects. (A)** Representative images of cells carrying *wild-type*, *priA*$^{tm}$*,* Δ*priB, or priA*$^{tm}$/Δ*priB* alleles (top: phase contrast, middle: SOS expression, bottom: nucleoids). Scale bar represents 5 µm. **(B)** Table listing quantification of cell area and SOS expression along with qualitative nucleoid partitioning phenotypes. Approximately 700-1500 cells were counted for quantification.

## Supplementary References


**1.** Heller, R. C. & Marians, K. J. Unwinding of the Nascent Lagging Strand by Rep and PriA Enables the Direct Restart of Stalled Replication Forks. *J. Biol. Chem.* **280**, 34143–34151 (2005).

2. Windgassen, T. A. & Keck, J. L. An aromatic-rich loop couples DNA binding and ATP hydrolysis in the PriA DNA helicase. *Nucleic Acids Res.* **44**, 9745–9757 (2016).

3. Sandler, S. J. *et al.* dnaC mutations suppress defects in DNA replication- and recombination-associated functions in priB and priC double mutants in Escherichia coli K-12. *Mol. Microbiol.* **34**, 91–101 (1999).

4. Leroux, M., Jani, N. & Sandler, S. J. A *priA* Mutant Expressed in Two Pieces Has Almost Full Activity in Escherichia coli K-12. *J. Bacteriol.* **199** (2017).

5. Windgassen, T. A., Leroux, M., Sandler, S. J. & Keck, J. L. Function of a strand-separation pin element in the PriA DNA replication restart helicase. *J. Biol. Chem.* **294**, 2801–2814 (2019).

6. Zieg, J. & Kushner, S. R. Analysis of genetic recombination between two partially deleted lactose operons of Escherichia coli K-12. *J. Bacteriol.* **131**, 123–132 (1977).

7. Tan, Y. Z. *et al.* Addressing preferred specimen orientation in single-particle cryo-EM through tilting. *Nat. Methods* **14**, 793–796 (2017).

8. Sagendorf, J. M., Berman, H. M. & Rohs, R. DNAproDB: an interactive tool for structural analysis of DNA–protein complexes. *Nucleic Acids Res.* **45**, W89–W97 (2017).

9. Landau, M. *et al.* ConSurf 2005: the projection of evolutionary conservation scores of residues on protein structures. *Nucleic Acids Res.* **33**, W299–W302 (2005).

10. Ponomarev, V. A., Makarova, K. S., Aravind, L. & Koonin, E. V. Gene Duplication with Displacement and Rearrangement: Origin of the Bacterial Replication Protein PriB from the Single-Stranded DNA-Binding Protein Ssb. *J. Mol. Microbiol. Biotechnol.* **5**, 225–229 (2003).
