# Supplementary material for: Replication fork binding triggers structural changes in the PriA helicase that govern DNA replication restart in E. coli
Source: Nat Commun. 2023 May 11;14:2725. doi: 10.1038/s41467-023-38144-x (PMC10175261; doi:10.1038/s41467-023-38144-x)
Supplement: Supplementary file 3 — Reporting Summary [file 41467_2023_38144_MOESM3_ESM.pdf]

nature portfolio

Corresponding author(s): Steven J. Sandler
Timothy Grant
James L. Keck

Last updated by author(s): Apr 12, 2023

Reporting Summary

Nature Portfolio wishes to improve the reproducibility of the work that we publish. This form provides structure for consistency and transparency in reporting. For further information on Nature Portfolio policies, see our Editorial Policies and the Editorial Policy Checklist.

Statistics

For all statistical analyses, confirm that the following items are present in the figure legend, table legend, main text, or Methods section.

n/a	Confirmed	
☐	☒	The exact sample size (*n*) for each experimental group/condition, given as a discrete number and unit of measurement
☐	☒	A statement on whether measurements were taken from distinct samples or whether the same sample was measured repeatedly
☒	☐	The statistical test(s) used AND whether they are one- or two-sided<br>*Only common tests should be described solely by name; describe more complex techniques in the Methods section.*
☒	☐	A description of all covariates tested
☒	☐	A description of any assumptions or corrections, such as tests of normality and adjustment for multiple comparisons
☐	☒	A full description of the statistical parameters including central tendency (e.g. means) or other basic estimates (e.g. regression coefficient) AND variation (e.g. standard deviation) or associated estimates of uncertainty (e.g. confidence intervals)
☒	☐	For null hypothesis testing, the test statistic (e.g. *F*, *t*, *r*) with confidence intervals, effect sizes, degrees of freedom and *P* value noted<br>*Give P values as exact values whenever suitable.*
☒	☐	For Bayesian analysis, information on the choice of priors and Markov chain Monte Carlo settings
☒	☐	For hierarchical and complex designs, identification of the appropriate level for tests and full reporting of outcomes
☒	☐	Estimates of effect sizes (e.g. Cohen's *d*, Pearson's *r*), indicating how they were calculated

Our web collection on statistics for biologists contains articles on many of the points above.

Software and code

Policy information about availability of computer code

Data collection	Electron Microscopy data was collected using SerialEM.
Data analysis	Electron Microscopy data was processed with CisTEM. Structural models were generated using ChimeraX, Coot, and Phenix. ImageQuant 10 was used to quantify all radio-imaged gels. GraphPad Prism 9 was used to analyze quantified gel data. Cells imaged by microscopy were segmented using Omnipose and Matlab was used to determine their relative fluorescence intensity.

For manuscripts utilizing custom algorithms or software that are central to the research but not yet described in published literature, software must be made available to editors and reviewers. We strongly encourage code deposition in a community repository (e.g. GitHub). See the Nature Portfolio guidelines for submitting code & software for further information.

Data

Policy information about availability of data

All manuscripts must include a data availability statement. This statement should provide the following information, where applicable:

- Accession codes, unique identifiers, or web links for publicly available datasets
- A description of any restrictions on data availability
- For clinical datasets or third party data, please ensure that the statement adheres to our policy

The cryo-EM map for PriA/PriB/DNA was deposited in the Electron Microscopy Data Bank (EMD-28959) and the final model was deposited in the Protein Data Bank (8FAK). Uncropped gel images and gel quantification data are provided in the Source Data file.

Human research participants

Policy information about studies involving human research participants and Sex and Gender in Research.

Reporting on sex and gender	Not applicable
Population characteristics	Not applicable
Recruitment	Not applicable
Ethics oversight	Not applicable

Note that full information on the approval of the study protocol must also be provided in the manuscript.

Field-specific reporting

Please select the one below that is the best fit for your research. If you are not sure, read the appropriate sections before making your selection.

☒ Life sciences ☐ Behavioural & social sciences ☐ Ecological, evolutionary & environmental sciences

For a reference copy of the document with all sections, see nature.com/documents/nr-reporting-summary-flat.pdf

Life sciences study design

All studies must disclose on these points even when the disclosure is negative.

Sample size	For Electron Microscopy, 1.5 million particles were used for 2D class averaging. ~200,000 particles were used for final 3D reconstruction refinements. For biochemical assays, three replicates were done for each experiment to ensure reproducibility. Genetics assays typically counted between 700 and 1500 cells for each strain. Sample sizes for structural biochemical, and genetic experiments were chosen to be consistent with other studies published in the field as well as previous studies published by the corresponding authors' labs.
Data exclusions	No data were excluded.
Replication	EM data was collected from multiple EM grids over several data collection sessions to achieve the dataset described above. All biochemical experiments were repeated three separate times. The results were repeatable and the data from all three replicates was averaged and plotted as shown in the manuscript with error bars. Genetics data were collected on hundreds to thousands of cells for each mutant.
Randomization	This is not applicable to this manuscript. The CryoEM, the biochemical, and genetics experiments reported cannot be randomized.
Blinding	This is not applicable to this manuscript. The CryoEM, the biochemical, and genetics experiments reported cannot be blinded.

Reporting for specific materials, systems and methods

We require information from authors about some types of materials, experimental systems and methods used in many studies. Here, indicate whether each material, system or method listed is relevant to your study. If you are not sure if a list item applies to your research, read the appropriate section before selecting a response.

Materials & experimental systems

n/a	Involved in the study	
☒	☐	Antibodies
☒	☐	Eukaryotic cell lines
☒	☐	Palaeontology and archaeology
☒	☐	Animals and other organisms
☒	☐	Clinical data
☒	☐	Dual use research of concern

Methods

n/a	Involved in the study	
☒	☐	ChIP-seq
☒	☐	Flow cytometry
☒	☐	MRI-based neuroimaging
